# Electro-Discharge Machining of Ceramics: A Review

**DOI:** 10.3390/mi10010010

**Published:** 2018-12-25

**Authors:** Azat Bilal, Muhammad Pervej Jahan, Didier Talamona, Asma Perveen

**Affiliations:** 1 Department of Mechanical & Aerospace Engineering, Nazarbayev University, Astana 010000, Kazakhstan; azat.bilal@nu.edu.kz (A.B.); didier.talamona@nu.edu.kz (D.T.); 2 Department of Mechanical Engineering, Miami University, Oxford, OH 45056, USA; jahanmp@miamioh.edu

**Keywords:** micro-EDM (electro-discharge machining), macro-EDM, ceramics, assistive EDM

## Abstract

Conventional machining techniques of ceramics such as milling, drilling, and turning experience high cutting forces as well as extensive tool wear. Nevertheless, non-contact processes such as laser machining and electro-discharge machining (EDM) remain suitable options for machining ceramics materials, which are considered as extremely brittle and hard-to-machine. Considering the importance of ceramic machining, this paper attempts to provide an insight into the state of the art of the EDM process, types of ceramics materials and their applications, as well as the machining techniques involved. This study also presents a concise literature review of experimental and theoretical research studies conducted on the EDM of ceramics. Finally, a section summarizing the major challenges, proposed solutions, and suggestions for future research directions has been included at the end of the paper.

## 1. Introduction

Structural materials such as ceramics, metals and polymers have their own advantages and disadvantages. Metals and polymers are not only suitable for high temperature and wear related applications but also are prone to chemical reactions. Retention of properties at higher temperatures is something that makes ceramic materials unique. On the other hand, ceramic materials outperform metals and polymers in terms of several characteristics. Due to the high hardness, higher strength, high temperature strength, high strength to weight ratio, biocompatibility [1], lower thermal & electrical conductivity, superior chemical stability as well as wear resistance, ceramics materials have become increasingly popular and have found their applications not only in the cutting tool industries but also in the biomedical and aerospace industries [2,3,4,5,6]. These properties meet the demands of the manufacturing of high quality microsystems as the mechanical components are constantly exposed to high temperatures and mechanical loads [7]. Biomedicine has a strong demand for tough and stable bioinert ceramics that is met by nano-structured ZrO_2_ or Al_2_O_3_. These ceramics are used for the fabrication of dental implants and restorations, acetabular cups, and femoral heads for total hip replacement, bone fillers and scaffolds for tissue engineering [8].

**Figure 1:**
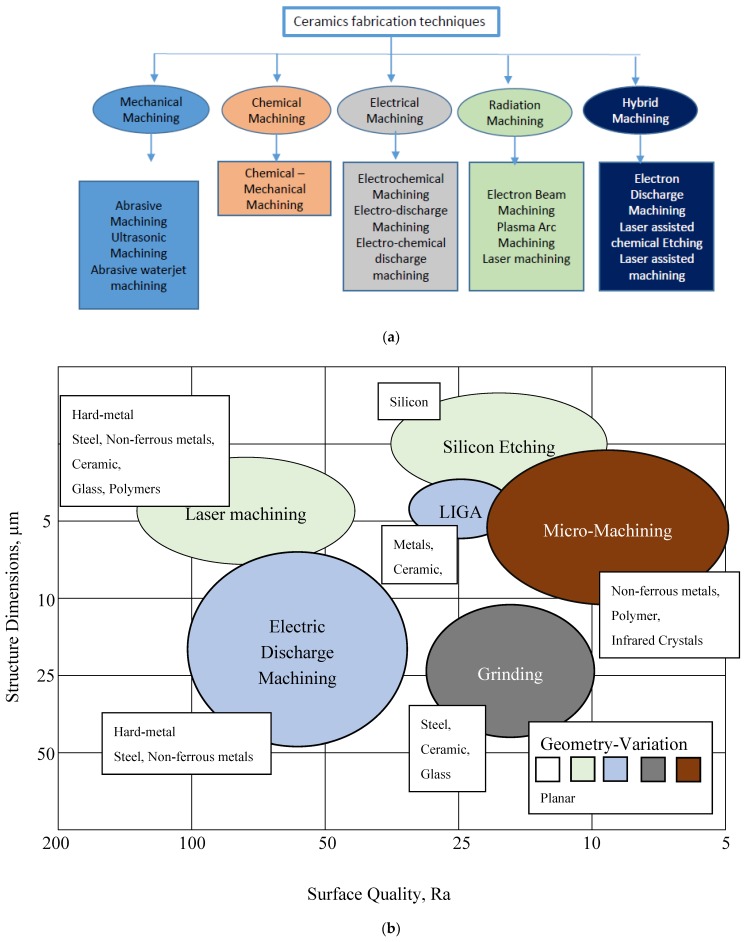
(**a**) Ceramic fabrication techniques [17]. (**b**) Electro-discharge machining (EDM) and micro-EDM relative to other machining processes.

**Figure 2:**
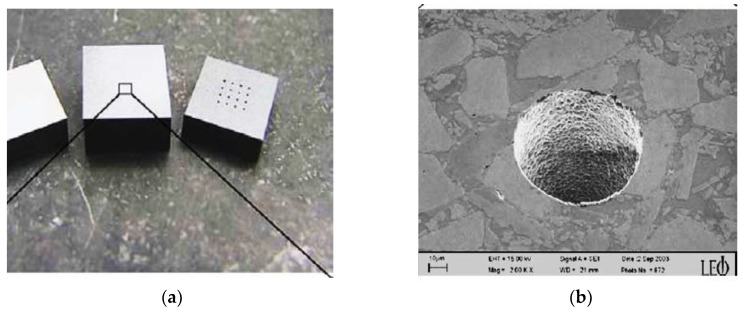
(**a**) Array of micro-holes in ceramic plates, (**b**) minimum hole diameter d_min_ = 80 µm in electrically conductive Si_3_N_4_. (Reproduced with permission from [51]).

**Figure 3:**
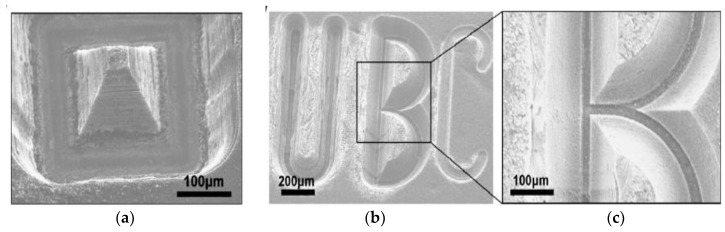
3-D μEDM of CNT forests using electrodes with cone-shaped tips performed at 35 V and 10 pF to form (**a**) a pyramid structure and (**b**) letters. Note the difference in the depth of the three letters U, B and C. (Reproduced with permission from [52]).

**Figure 4:**
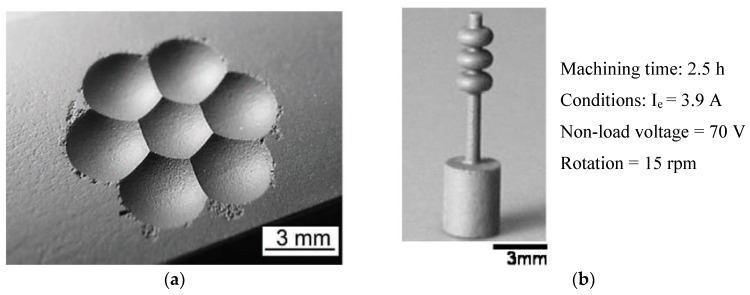
(**a**) Die for fly-eye lens machined by EDM in Si_3_N_4_ ceramics. (Reproduced with permission from [53]) (**b**) small product on Silicon nitride using Wire EDM. (Reproduced with permission from [54]).

Ceramic materials can be conductive, semi-conductive and non-conductive. Some examples of commonly used ceramics are alumina, aluminum nitride, boron carbide, silicon nitride, zirconia, sialon, titanium carbide, titanium nitride, zirconium nitride, and silicon carbide, etc. [9]. Properties and applications of some ceramics are provided in the Table 1. Nevertheless, the properties that have made ceramic materials one of the most desirable engineering materials also hinder their machining characteristics. The main drawback of ceramics lies in their cost and the complex manufacturing cycle, essentially during the finishing step. For conventional machining of hard ceramic components, the limitation is in net-shape fabrication and hence there is requirement to additionally machine ceramics with low materials removal rate (MRR). In this case, low material removal rate makes the application of ceramic components uneconomical, [10] as the cost of the final parts increases. The challenges in processing these materials are due to the large cutting forces and high tool consumption. Therefore, the greatest challenge that exists in achieving the full potential of ceramic materials is to develop material removal techniques without compromising the ceramics’ properties, as well as developing techniques that offer both an economical solution and less residual damage. Figure 1a shows different techniques for the fabrication of ceramics materials. Most of the conventional mechanical machining techniques, such as milling, turning [11], drilling, grinding, find it impossible or challenging to machine ceramics and also suffer from extreme tool wear associated with high cutting forces, as well as chatter [12] long process time and higher expenses. Therefore, mechanical machining processes seem to be not feasible for ceramic fabrication techniques [9]. Considering its contributions to environmental hazards, chemical machining [13,14] is not considered as an environmentally friendly process. Other non-contact processes such as laser machining [15,16,17,18], plasma machining [19,20], electron beam machining [21,22,23], electrochemical machining [24,25,26,27], ultrasonic machining [28,29,30,31,32], and abrasive water jet machining [33,34,35,36,37] also have their advantages and disadvantages as reported by different researchers. Therefore, non-contact machining processes have received more attention considering they employ no-force machining as well as recognize the brittleness of the ceramic materials. One such non-contact machining process is the electro discharge machining process (EDM), which has revolutionized the machining process in the way it removes materials. Figure 1b presents the relative contribution of EDM and micro-EDM compared to other machining techniques for surface and dimension generation. EDM is an electro thermal process that uses discharge energy to remove materials and is not dependent on materials’ hardness properties [38,39,40]. However, EDM requires the material to have a certain level of electrical conductivity. Even though conductive ceramics can be eligible to be machined by the EDM process, semi-conductive as well as non-conductive ceramics represent a considerable amount of challenges for the EDM process. EDM process has evolved to face these challenges and therefore currently, assistive EDM makes it possible to machine even non-conductive ceramic materials. It is worth mentioning that the manufacturing of micro-ceramics parts includes the machining of a large number of microstructures such as holes, channels, surfaces, and recesses in high-density ceramics. For this, industry development of EDM processes for micro-machining of semi-finished products made of high density ceramics is essential [41]. It is noted that among the applications of EDM, there is a need for manufacturing of cooling holes in the turbine blades to create a thin film of cooling layer, and components for fuel injection and filters. Due to mass production, these components require quality, high efficiency and environmental protection [7]. Another application of EDM found in the drilling of micro-holes, which are widely used in industry. Common examples include spinnerets holes, inkjet printer nozzles, blades cooling channels, drug delivery orifices, and Diesel fuel injection nozzles [42]. Figure 2, Figure 3 and Figure 4 present some examples of microstructures machined by EDM.

Currently, there is a high demand for environmentally friendly and safe manufacturing processes. This has become a goal of many companies, especially after the introduction of the ISO 14000 standard of the environmental management system [7]. Considering that EDM has a small environmental impact, this process can be improved to a fully environmentally friendly manufacturing process by eliminating the liquid from the process [43]. Considering the significant importance of ceramics machining, this paper aims to present a systematic review of ceramics machining processes, fundamental of EDM processes and experimental investigation of ceramics machining using an EDM process.

## 2. Ceramic Materials

### 2.1. Conductive

**Figure 5:**
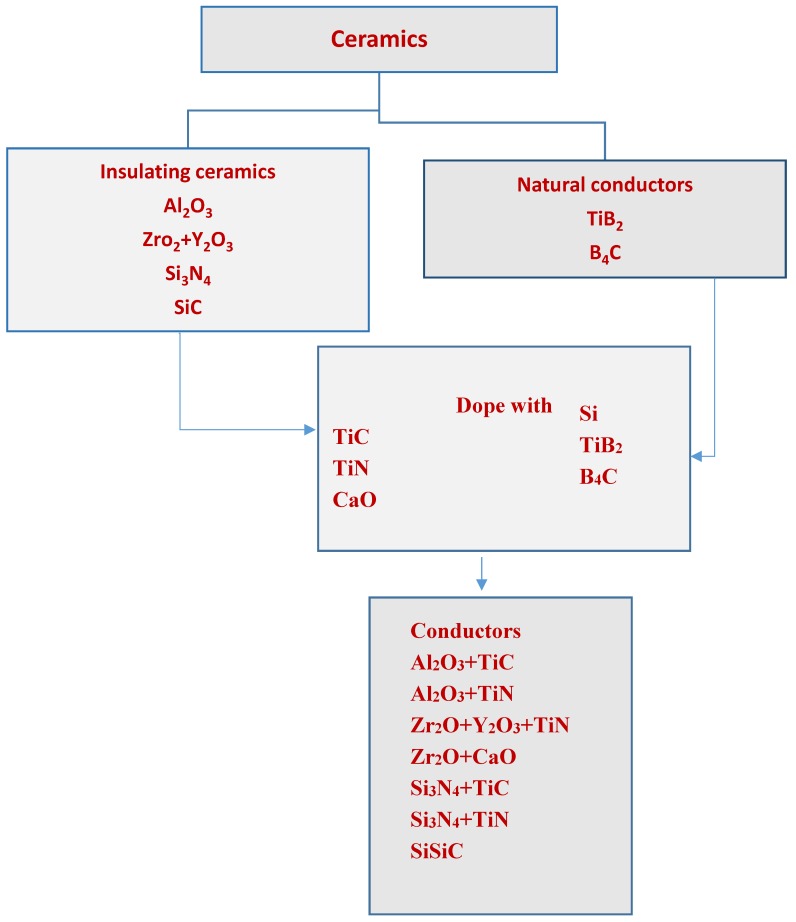
Classification of ceramics materials based on conductivity. (Reproduced with permission from [61]).

Figure 5 shows the classification of ceramics based on their conductivity values. The minimum electrical conductivity required for electrical discharge machining is about 10^−2^/Ωcm [55]. All metallic materials fulfil this demand. However, for ceramic materials possessing minimum electrical conductivity, this becomes a crucial factor when it is to be machined by EDM.

**Figure 6:**
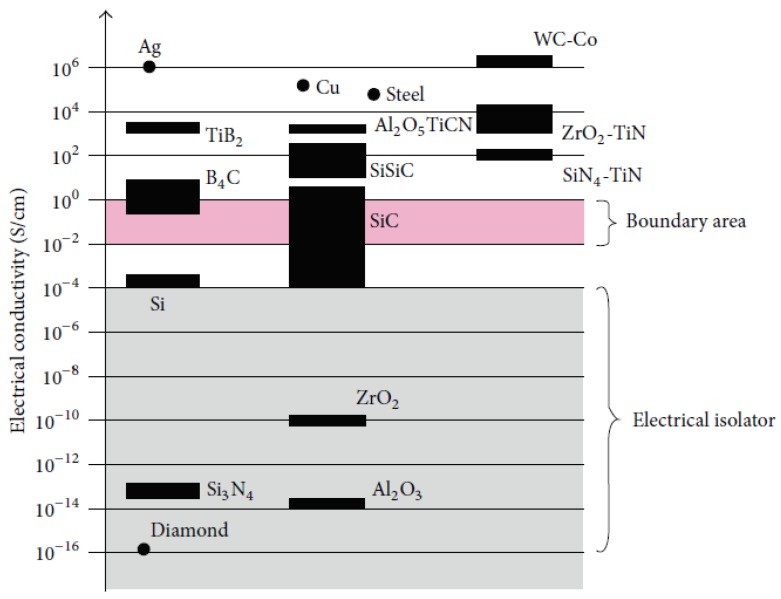
Electrical conductivity of materials [55].

As can be seen from Figure 6, electrically conductive ceramics such as titanium nitride (TiN), titanium diboride (TiB_2_) or silicon doped silicon carbide (SiSiC) can be easily machined by EDM, which is similar to other metallic materials [56]. Nevertheless, nonconductive ceramics such as aluminum oxide (Al_2_O_3_), silicon nitride (Si_3_N_4_), or zirconia (ZrO_2_) do not meet the minimum requirement of electrical conductivity needed for EDM (10^−2^/Ωcm). To influence the electrical conductivity of insulating ceramics such as Al_2_O_3_, Si_3_N_4_ and ZrO_2_, a secondary electrically conductive phase is incorporated into ceramics, which allows machining by EDM. A successful approach was achieved by Yoo et al. (2015) with the SiC ceramic reinforcement by yttrium nitrate (YN). For Si_3_N_4_, the incorporation of TiN has been reported as successful [57]. However, such an approach has one disadvantage, in that reinforcements can influence mechanical properties of the material. For example, increasing the TiN content will have a negative effect on hardness, fracture toughness and flexural strength of ZrO_2_ [58]. Regarding the fatigue limit of conductive ceramics such as SiC fiber aluminosilicate reinforced composite, it was found to be 50% below the monotonic proportional limit strength for 25,000 cycles at room temperature [59]. For another conductive co-continuous metal-ceramic, the fatigue limit was 91 MPa at room temperature for 10^7^ cycles [60].

### 2.2. Non-Conductive

For non-conductive ceramics such as Al_2_O_3_ (aluminum oxide), ZrO_2_ (zirconia) or Si_3_N_4_ (silicon nitride), the electrical conductivity is very low so as to allow the electrical current to pass between workpiece and tool electrode. However, there is a way to machine non-conductive ceramics without reinforcing it. This method is called the ‘Assisting Electrode Method’ [62]. Non-conductive alumina (Al_2_O_3_) is the most commonly used oxide ceramics that has moderate to high strength, and is usually used to make abrasives, cutting tools, and thermal and electrical insulator. Another non-conductive ceramic zirconia (ZrO_2_) has high toughness and strength, and its thermal expansion is approximately close to cast iron and finds its application in jet-engine components, high temperature applications, and nuclear fuel cladding. Silicon nitride (Si_3_N_4_) has a high thermal and creep resistance. Common applications of Si_3_N_4_ include rolling element bearing, turbocharger components, and cutting tools [63]. Regarding the fatigue of the ceramics, it has been found that the mechanism of fatigue failure is different from metals, which involves plastic deformation. The physics of its occurrence in ceramics is related to the degradation of toughening elements in the microstructure. For non-conductive ceramics, such as zirconia having medium strength, the fatigue limit for 10^8^ cycles at room temperature is approximately half of single-cycle fatigue strength; whereas, alumina at the same conditions fails at a stress of 25–40% of the single-cycle strength [64].

From the Table 2, it can be noticed that transverse rupture strength for various ceramics are about one order of magnitude less than the compressive strength. This is because of the ceramics’ high sensitivity to impurities, cracks, and porosity. Under tensile stress, these defects result in crack initiation and propagation; and also under static tensile load, ceramics suddenly fail. This usually happens in the environment with water vapor present [63]. Ceramics usually lack thermal-shock resistance and toughness due to poor ductility. Thermal conductivity and thermal expansion lead to internal stresses that can result in thermal fatigue or thermal shock. However, by combining high thermal conductivity and low thermal expansion, the tendency for thermal cracking can be decreased. The optical properties can be varied in many ways by controlling the structure. In addition, porosity has s strong influence on the optical properties of ceramics. Because of trapped air, porosity makes ceramics less transparent [63].

## 3. Principle of EDM

### 3.1. Basic Mechanism of EDM/Micro-EDM

**Figure 7:**
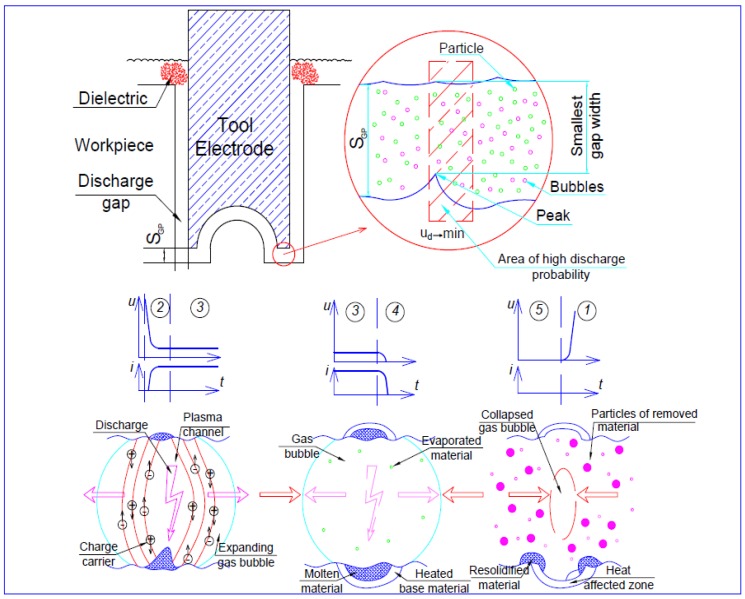
Machining principles of EDM [55].

EDM is a process of removing electrically conductive materials by means of rapid and repetitive spark discharges that occur between the tool electrode and the workpiece at the presence of dielectric oil. During the EDM process, a conductive tool electrode with the desired geometry is brought to close proximity of the workpiece by maintaining a small gap known as a spark gap. Both the workpiece and electrode, especially the spark gap zone of the electrode and the workpiece, are submerged in the dielectric fluid. The electrical voltage is applied between the electrode and the workpiece, which results in a series of sparks causing the breakdown of the dielectric, which in turns results in an intense amount of heat, eventually melting and evaporating the materials from both the workpiece and the electrode (Figure 7). The dielectric flushing is used to remove the machined craters from the spark gap, and to draw fresh dielectric into the spark gap for the next series of sparks to take place. The basic mechanism of material removal in EDM is similar to the micro-EDM process, but with significant differences in the process mechanism, capabilities, and machine tools used for the processes. The physics of various phenomena happening during the EDM process is discussed in the next section.

### 3.2. Sparking and Gap Phenomena in EDM/Micro-EDM: Physics of the Process

Figure 7 illustrates the schematic diagram showing the basic principle of the EDM process, as well as the sparking and gap phenomena during the machining process. An electric discharge is realized by applying a voltage between tool and workpiece while the distance between these electrodes is decreased. When the breakdown voltage of the dielectric medium is reached, discharge occurs, which allows the establishment of a plasma channel through which current flows [66]. This leads to a temperature rise of more than 10,000 K, which in turn, results in material melting and evaporation, creating a rapidly expanding gas bubble. After stopping the energy input, the plasma channel collapses and therefore the discharge ends. The gas bubble formed earlier also collapses. With the aid of this collapse, the flow of the dielectric medium flushes the particles of the removed material away. Removal of material occurs by the repetition of this process and because of the tool feed in the workpiece direction; its shape is imprinted on the workpiece material. The wear of the tool also exists due to the thermal influence on both electrodes. The surface resulting after this process is described by an assembly of single discharges, which results in a crater-like topology. The main factor on which the crater dimension depends is the applied discharge energy and this can be controlled by choosing respective process parameters. The discharges occur at the region where dielectric breakdown voltage is the lowest. Usually, the lowest dielectric breakdown voltage is in the area where the distance between electrode and workpiece is the smallest. However, discharges also may take place in regions with a larger distance between workpiece and tool because of the presence of removed material particles and gas bubbles, which reduce the dielectric fluid’s breakdown voltage. Because of the thermal ablation of the workpiece, this process does not depend on the hardness and brittleness of the material, which is the main advantage of electrical discharge machining. Additionally, the use of fragile and thin tool electrodes to machine small geometries with high aspect ratios by using nearly force-free processing can be utilized [67].

### 3.3. Differences between EDM and Micro-EDM

Even though the physical principles of the micro-EDM are similar to those of the macro-EDM and both use spark erosion, micro-EDM is not just an adoption of the EDM to micron level. There are significant differences in the size of the tool used, the fabrication method of micro-sized tools, the power supply of discharge energy, movement resolution of machine tools’ axes, gap control and flushing techniques, and in the processing techniques [68,69,70]. The most important difference between macro-EDM and micro-EDM is the dimension of the plasma channel. For the macro-EDM, the size of the plasma channel is much smaller than the electrode size, while the size is comparable for micro-EDM [71]. Lee et al. [72] suggested secondary, thermionic and field emission as the causes behind the emission of electrons from the cathode during micro-EDM. Under the conditions of EDM discharges, where there exists strong temperature and electrical fields, according to the Thomas–Fermi theory, the emission process is greatly dependent on both fields [73]. Because of small electrodes, the maximum energy that can be reached is limited in micro-EDM, since excessive discharge energy can lead to electrode burn (or wire rupture in wire-EDM) [74]. As a result, for each discharge, the electrode wear in micro-EDM is proportionally higher than conventional EDM. Also the flushing of debris is more difficult in micro-EDM because of small gap size, high dielectric viscosity, and a higher pressure drop in micro-volumes [75].

**Figure 8:**
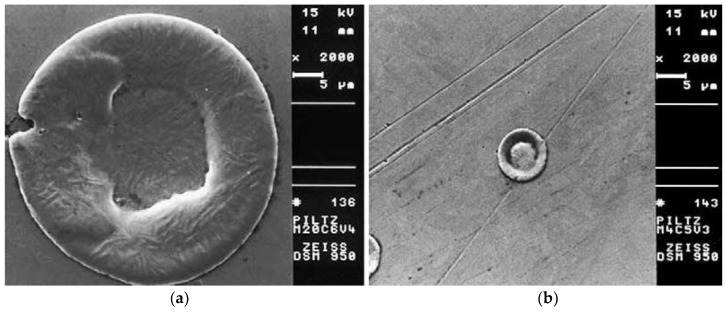
Comparison between crater dimensions in (**a**) conventional EDM (left) and (**b**) micro-EDM (right) (Reproduced with permission from [51]).

The micro-EDM process requires small energies of 10^−6^–10^−7^ J for every discharge of 40–100 V and high frequencies of greater than 200 Hz [75]. For micro-EDM, discharge durations of less than 1 µs and discharge energies of less than 100 µJ are common. This allows the machining of geometrical features with diameter of less than 5 µm and a depth of less than 1 µm. In such conditions, the achievable surface roughness is lower than 1 µm [55]. The precision and accuracy of the final products are much higher in micro-EDM [76]. The crater sizes in micro-EDM are also much smaller than that of conventional EDM [76]. Figure 8 shows the comparison of the crater size between conventional EDM and micro-EDM [51].

In a study using a RC (Resistance-Capacitor) type pulse generator to investigate the micro-EDM process mechanism, Qian et al. [77] reported on the occurrence of resonance with a large amplitude and frequency in the pulse generator circuit consisting of parasitic inductance, charging capacitance, parasitic capacitor of the discharge gap and discharge cable and gap voltage. High frequency sparks cause incomplete neutralization of the developed plasma channel due to insufficient time availability, and this in turn causes gap breakdown under reverse polarity due to the availability of negative voltage as well as debris. This ultimately initiates alternating current and the material removal rate improves as compared to the case when negative current is non-existent. Another study by Wong et al. [69] compared material removal rates obtained by conventional and micro-EDM processes by using a single spark generator. They reported on the lower specific material removal energy for low discharge energy compared to high discharge energy and consistently uniform craters for small discharge energy. Ghosh et al. [78] reported on the different removal mechanisms due to different pulse duration. For a pulse duration of less than 5 µs, dominant material removal is not due to melting but due to large electrostatic forces. A very short pulse duration does not provide enough time to adequately heat the surface to reach its melting or evaporating points. Therefore, the material removal mechanism associated with micro-EDM depends on the complex mechanism, and hence, the thermal modeling of the material removal mechanism alone may not be sufficient.

## 4. EDM of Conductive Ceramics

The EDM of ceramic materials exhibits certain distinctive characteristics. Compared to metallic materials, ceramics demand higher energy for the removal of material by melting and vaporization as well as to overcome higher electrical resistance. In addition, thermal stress generated material removal mostly occurs during the EDM of ceramics. The choice of pulse frequency is limited, which is governed by the electrical resistance of the ceramics [79,80]. The EDM of the ceramics generates thermal energy in terms of a spark similar to metallic materials. However, the conductive area is dispersed in the ceramics instead of a uniform distribution, as is the case for metal. When the appropriate potential is applied, the magnetic field appears between the conductive tool and the conductive areas of the ceramics which are separated by dielectric. Eventually the higher energy plasma channel build up breaks the dielectric strength and reaches a temperature as high as 40,000 K, which causes vaporization [81].

**Figure 9:**
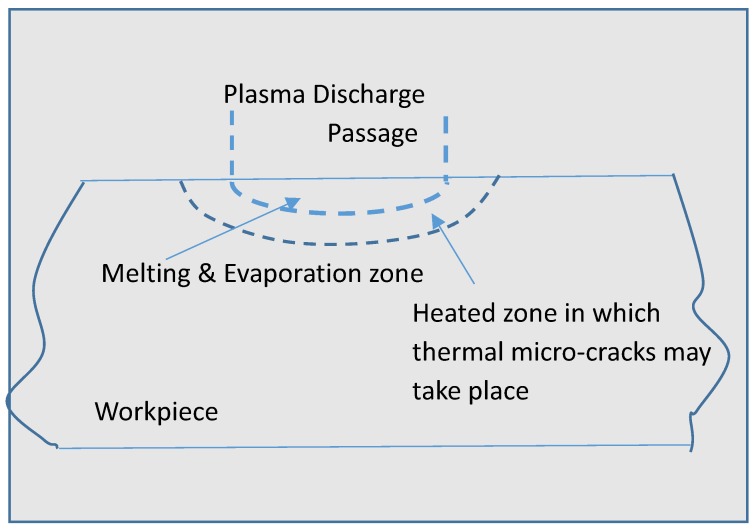
Thermal spalling effect on ceramic. (Reproduced with permission from [79]).

For EDM with metallic materials, removal mechanism is dominated by electromagnetic and electrostatic force when the pulse duration is less than 1 µs. However, in the case of a longer pulse duration; melting, vaporization, as well as thermal spalling dominate the removal mechanism. The proportion of materials removed by both mechanisms largely depends on the process parameters and physical as well as mechanical characteristic of the materials. As suggested by Lee et al., the EDM removal of ceramics (Al_2_O_3_ + TiC) is not only due to the effects of melting, evaporation, and oxidation but is also due to thermal spalling. In addition, thermal spalling causes the removal from the deeper level of the workpiece compared to the usual melting and as well as evaporation, these contribute to high surface roughness as shown in Figure 9. The brittleness of ceramics along with lower thermal and electrical conductivities initiate the establishment of an abrupt temperature gradient away from the melt and vaporizes the area, causing thermal spalling [82,83]. For ceramics, thermal spalling plays the dominant role, which is governed by the physical and mechanical behavior of ceramics. Sometimes, dominant thermal spalling pairs up with electro discharge build up (EDBU), which is not desirable [79].

**Figure 10:**
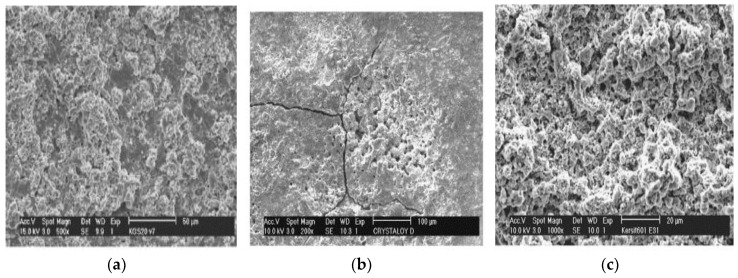
(**a**) Topography of ZrO_2_–TiN by wire EDM (V = 120 V, pulse on = 2.4 µs, pulse off = 15 µs); (**b**) Topography of Al_2_O_3_–SiCW–TiC by die sinking EDM in oil (high energy) (V = 250 V, i = 72 a, pulse on = 7.5 µs, pulse off = 18 µs); (**c**) Topography of Si_3_N_4_–TiN by Wire EDM (V = 160 V, pulse on = 2.8 µs, pulse off = 2.3 µs). (Reproduced with permission from [84]).

Lauwers et al. [84] investigated the material removal mechanism associated with ceramics composites such as ZrO_2_-based, Si_3_N_4-_based and Al_2_O_3_-based ceramic materials using die sinking as well as wire EDM by analyzing debris and surface/subsurface conditions. They suggested three mechanisms called, thermal spalling, melting/oxidation and melting/evaporation for material removal of these kinds of ceramics using EDM. Their investigation on zirconia ceramics using wire cut EDM reveals the probable material removal mechanism involved to be melting/evaporation due to the presence of several droplets on the machined surface as shown in Figure 10a. In addition, there exists micro-cracks on the recast layer found in the cross section of the machined surface. The second mechanism spalling involves material removal by separation of small volume from bulk material, which is mostly due to the formation of large cracks propagating both in the perpendicular and parallel directions. Die-sinking EDM of Al_2_O_3_–SiCW–TiC at high-energy condition exhibits this kind of material removal mechanism as seen from Figure 10b. In addition, die sinking EDM of Si_3_N_4_–TiN also suggests a similar spalling mechanism. Debris generated during the process is irregular in shape, containing both round and sharp edges, confirming the removal mechanism by spalling. On the other hand, wire EDM of Si_3_N_4_–TiN shows both a foamy and porous layer as shown in Figure 10c. Foamy microstructures are due to the formation of gas bubbles, as confirmed by the visual presence of bubbles and the smell of ammonia during the experimentation. Thermal energy generated during the EDM process contributes in oxidation/decomposition of ceramics, which eventually leads to these effects. Release of generated gas also results in a porous structure in the machined surface/subsurface.

Lee et al. [71] conducted die-sinking EDM on Al_2_O_3_ + TiC ceramics using copper electrode. Their observation suggested the development of EDBU due to the adherence of ceramics particles toward the electrode under certain process conditions. During normal EDM conditions, the eroded particles go either to the gaseous phase or liquid phase and they can be easily flushed by dielectric due to their minute size as well as the charge carried. However, during a reverse charge condition, workpiece material will be broken away by large particles carrying a similar charge as the parent material and thus depositing on the electrode carrying an opposite charge. Eventual discharge from the electrode will be concentrating on this area to help build EDBU. EDBU is influenced by pulse energy, pulse on, and pulse off time apart from dielectric type. Both larger pulse energy and longer pulse duration assist in EDBU formation. Insufficient pulse off time as well as a positive terminal electrode also aids EDBU. However, pressurized flushing can help reduce this effect [79]. This EDBU phenomena is also demonstrated by other researchers as well [85,86]. With the proper process condition, and by avoiding EDBU, Lee et al. reported on the surface roughness of 4 µm and removal rate of 0.6 mm^3^/min using die-sinking EDM. Puertus et al. [87] conducted a comparative die-sinking EDM study on cobalt-bonded tungsten carbide, hot-pressed boron carbide and reaction-bonded silicon carbide. According to their observation, increased pulse on time increases the surface roughness of all the ceramics, but an increased duty cycle increases the roughness for all the ceramics except boron carbide. Again, an increase of pulse on time has a decreased effect on the electrode wear in the case of boron carbide, whereas it is opposite for the others. In addition, an increase of the duty cycle causes increasing electrode wear for both boron carbide and silicon carbide. Moreover, increased pulse on time also reduces the MRR for both SiC and WC. Furthermore, increased duty cycle contributes to increasing the MRR for all the ceramics. A similar study on Silicon carbide ceramics was reported by Noble et al. [88]. Another study by Puertas et al., conducted a statistical investigation on boron carbide to optimize performance parameters such as MRR, electrode wear ratio (EWR), and surface roughness using the EDM process [89]. A similar study on Siliconizes silicon carbide was also conducted by Puertas et al. and they reported on the mathematical modeling of performance parameters in terms of process parameter such as pulse on time and duty cycle [90]. Sa´nchez et al. [91] demonstrated and optimized die-sinking EDM and wire EDM processes for B_4_C and SiSiC materials. In the case of wire EDM, roughness as small as 0.56 µm for boron carbide and 3.5 µm for silicon infiltrated silicon carbide were reported by Sa´nchez et al. Die sinking EDM can achieve a MRR of 8.3 mm^3^/min for B_4_C and 10.1 mm^3^/min for SiSiC whereas it is possible to reach roughness below 1 µm provided no capacitor is used. Lok et al. investigated on SiAlON and Al_2_O_3_-TiC, using wire-EDM and reported on the MRR and surface roughness. According to their investigation, volumetric MRR is inferior compared to metal, as well, the roughness achieved by wire electrical discharge machining (WEDM) is much higher when compared with die-sinking EDM. Their study also reported on the drop of flexural strength to 67% for SiAlON and 32% for Al_2_O_3_-TiC, which may be partly due to thermal damage as well as generation of intrinsic cracks [92,93]. Another study, which considered the modelling and process parameters optimization for EDM of tungsten carbide-cobalt composite, also planned the machining mode using statistical techniques [86]. To plan and analyze the experiment, response surface methodology (RSM) was used. It was revealed that by selecting a higher duty cycle and discharge current resulted in an increase of MRR, which occurred because greater discharge energy was provided inside the gap region. While applying the longer pulse on time, tool wear can be reduced by lower current intensities, whereas a smoother surface can be obtained with small pulse durations when a relatively high discharge current is used to get a more effective discharge and better plasma flushing [94]. In the study of Patil & Brahmankar (2010) [95] a semi-empirical model of MRR in WEDM based on the machining parameters including average gap voltage, pulse on time, and thermo-physical properties of the workpiece were presented. The development of model was achieved by using quasi-Newton and simplex. The workpiece material was a silicon carbide particulate reinforced aluminum matrix composite. The model prediction and experiments show that coefficient of thermal expansion plays a significant role in the wire EDM of this material. The response surface method was used to create the empirical model, which shows good agreement of these models with predictions; however, the agreement was limited to a low pulse on time (0.2 µs). Moreover, the increase of 10% in ceramic reinforcement resulted in a 12%decrease of MRR.

**Figure 11:**
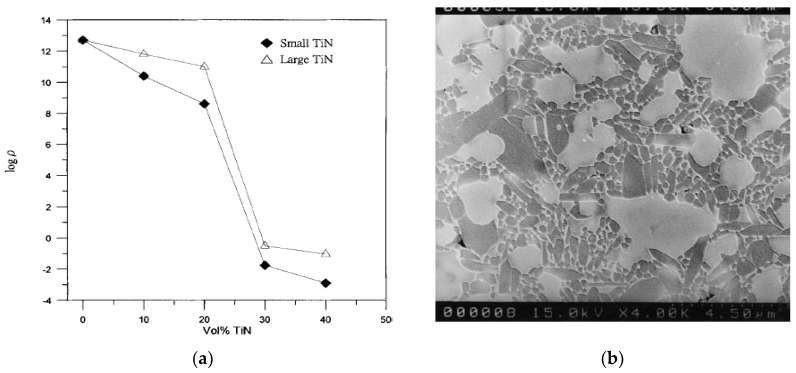

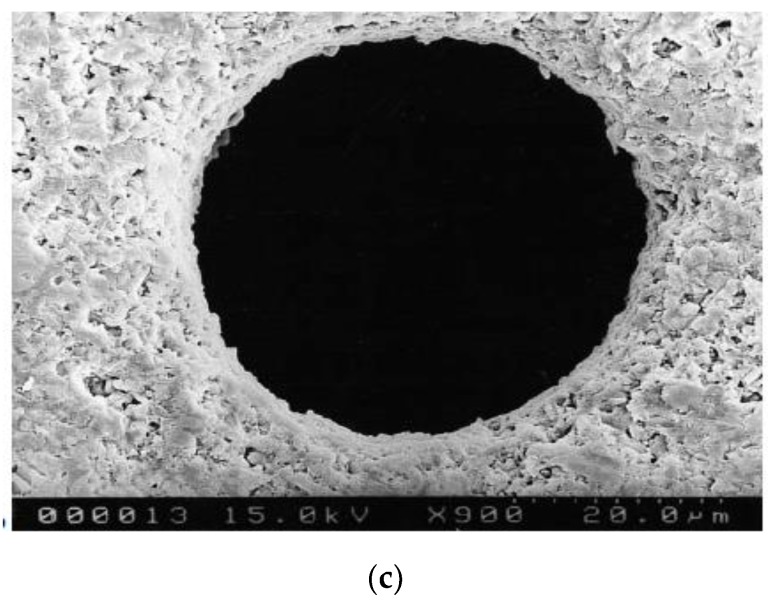
(**a**) Electrical resistivity of TiN/Si_3_N_4_ composites as function of TiN content (**b**) 40 vol. % small TiN/Si_3_N_4_; large white areas represent TiN while gray regions indicate Si_3_N_4_ (**c**) 700 µm depth and 70 µm dia. hole machined by Micro EDM. (Reproduced with permission from [97]).

Electrical conductivity of nonconductive ceramics can be enhanced with the incorporation of a conductive phase into the ceramic matrix, such as (TiN), borides (TiB_2_, ZrB_2_), and carbides (TiC), so that successful EDM operation can be conducted [96]. Liu et al. successfully demonstrated micro-EDM of one of such composite TiN/Si_3_N_4_ where; 40% volume of TiN addition has decreased the electrical resistivity value to 1.25 × 10^−3^/Ωcm. Figure 11a,b show the electrical resistivity value distribution with TiN content, and microstructure of the composite after TiN incorporation [97]. Figure 11c shows the micro holes machined on TiN/Si_3_N_4_ by the micro-EDM process successfully. Nevertheless, the size and volume of TiN has a significant impact on the fracture toughness and flexural strength of the ceramics. As the volume of the conductive phase increases, toughness also increases, compromising the strength properties of ceramics. On the other hand, large size TiN enhances the fracture toughness while compromising flexural strength compared to the small particle. A similar study on Al_2_O_3_ + 30% TiC, Al_2_O_3_ + 40% TiC, TiB_2_, SiSiC, SiAlON were carried out by Dauw et al. using WEDM and reported on the materials properties’ dependency on EDM performance unlike the EDM of metallic materials [98,99].

**Figure 12:**
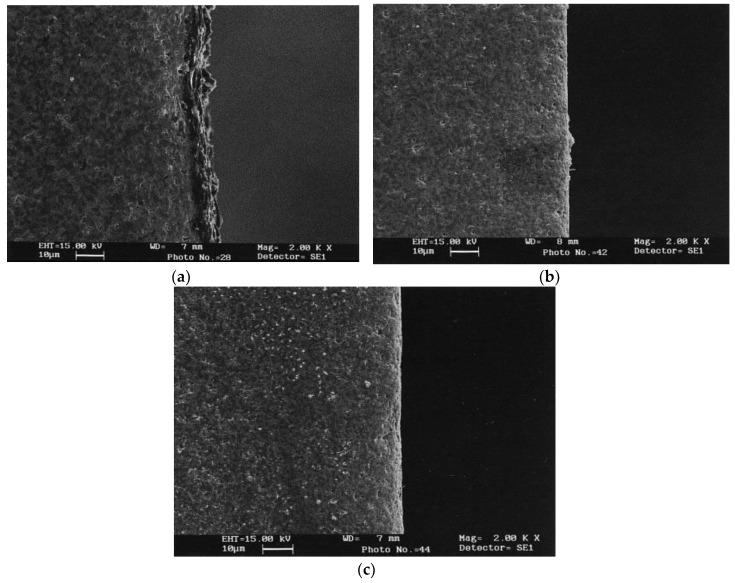
Cross-sectional scanning electron microscope (SEM) micrographs of EDM and treated surfaces of Al_2_O_3_/TiC ceramic composite (**a**) EDM; (**b**) treated by abrasive blasting; (**c**) treated by ultrasonic machining. (Reproduced with permission from [107]).

Matsuo et al. investigated Zirconia incorporated with 28–45% NbC and TiC using wire EDM and reported on the optimum machining conditions. According to their investigation, 28–30% volume of conductive phase provides the maximum machining rate and the increase of the carbide content in general, increases the surface roughness. Lower carbide content provides a reduced machining rate due to the inadequate discharge energy as a result of the low electrical conductivity [100]. Zhang et al. investigated hot pressed alumina containing TiC and WC using EDM and stated the possibility of using the product of thermal conductivity and fusion temperature as an indication of the machinability index for ceramic materials. According to their investigation, with the increase of pulse on time (10 to 1800 µsec) MRR, roughness, discharge point diameter show an increasing trend. While discharge current has an imperative impact on MRR as well as a discharge point diameter, pulse on time mostly affects roughness and the heat affected zone [101]. Pitman et al. [102] investigated ZrO_2_ ceramics incorporated with TiN using a die-sinking EDM. Their study reported on maximum volumetric MRR of 4 mm^3^/min, relative volumetric tool wear of 0–5% with the process condition of sparking duration of 50 µs, a current of 48 A, and a duty factor of 11.1%. Their observation about a material removal mechanism involves spalling large particles because of rapid temperature increment leading to subsurface cracks generation and then subsequent rapid cooling. Rapid cooling is accompanied by the phase transformation in the ceramic causing the generation of quench cracking. Finally, the cracks generated propagate circumferentially towards the direction of the maximum stress field due to a large temperature gradient and higher tensile stress. On another surface characteristic investigation by Ramulu et al. [103], silicon carbide ceramics impregnated with TiB_2_ (20%) was machined with the help of brass, copper, and graphite electrodes. Their results suggested the dependency of surface quality not only on the EDM process parameters but also on the electrode materials. Subsurface damage generation during the EDM are found to be associated with the thermal cracking and chipping, which leads to a reduced hardness value. However, the damage layer and cracks extend less than 1 µm below the surface during fine finishing condition. Ramulu et al. [104] also investigated the machinability of Sic-TiB_2_ against mild steel using die sinker EDM and reported on the efficient machinability of ceramics under very slow cutting conditions. A similar study was also conducted on 15 and 25 vol % SiC whisker-2124 aluminum composites and TiB_2_/SiC [105,106]. Ceramics surface machined by the EDM process in general contain a damage surface layer as well as cracks, which can be removed either by subsequent ultrasonic or abrasive blasting processes in order to enhance the surface integrity, strength and Weibull modulus. Deng et al. investigated Al_2_O_3_/TiC and Si_3_N_4_/TiC ceramics where the EDM process was followed by ultrasonic or blasting finishing processes for enhancing the surface integrity. They reported on the decreased hardness and increased roughness after the EDM process and the enhanced hardness as well as reduced roughness after ultrasonic or blasting process [107]. Figure 12 shows the cross-sectional view of surface after every process. According to their results, EDM machined surface provided strength of 538 MPa, which was enhanced by abrasive blasting to 541 MPa, and was further enhanced by ultrasonic finishing to 583 MPa. In addition, the value of the Weibull modulus increased from 7.4 (EDM) to 10.5 (abrasive blasting) to 17.5 (ultrasonic machining).

**Figure 13:**
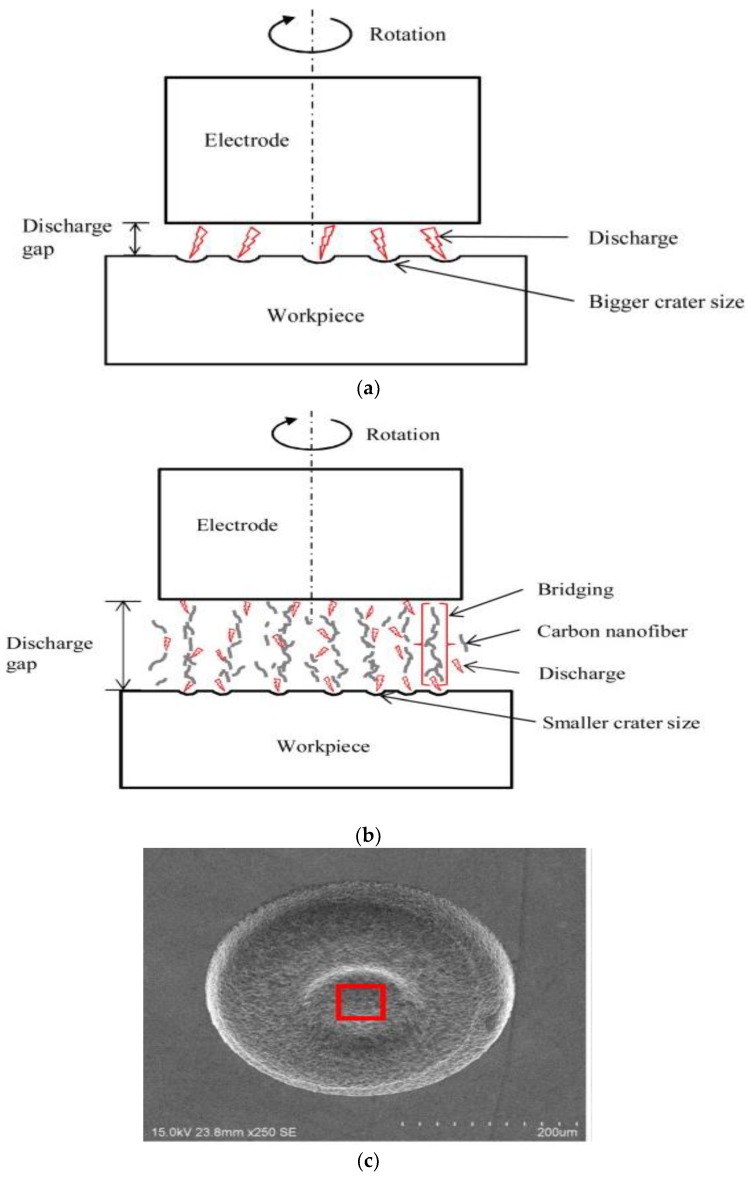
Schematic model for (**a**) conventional EDM and (**b**) carbon nanofiber assisted micro EDM, (**c**) machined surface at machining depth of 20 μm with 0.06 g/L carbon nanofibers. (Reproduced with permission from [47]).

Lopez et al. [108] also demonstrated the potential of adding a semi-conductor or metallic phase to the ceramic materials in order to enhance the electrical as well as the mechanical properties of ceramic composites. Their study demonstrated EDM of zirconia composites (ZTN) due to the presence of a metallic/semi-conductive phase. Their observation suggested that either the nanoparticle reduces the breakdown voltage for the deposited layer, thus allowing continuous EDM or due to the low ionization potential of Ni compared to TiC, addition of nickel component aids in generating the plasma channel. Luis et al. conducted die-sinking EDM of siliconized silicon carbide (SiSiC) using fractional (2^5−1^) factorial design in order to find the main effect, interaction effect, and quadratic effect of process parameters such as pulse on time, intensity, duty factor, voltage, and flushing pressure. Their investigation suggested that the intensity and voltage are the main parameters to affect the MRR whereas intensity, pulse time and flushing pressure are the main parameters to affect the electrode wear [109]. Liew et al. [47] demonstrated carbon nanofiber (CNF) assisted micro-EDM for reaction-bonded silicon carbide using a RC type pulse generator. A comparison between conventional and carbon nanofiber assisted micro-EDM are illustrated in Figure 13a,b. In carbon nanofiber assisted micro-EDM, the carbon nanofibers form micro-chains bridging the tool electrode and workpiece by organizing themselves automatically, as shown in Figure 13b. Due to the excellent electrical conductivity of CNF, breakdown strength of the dielectric reduces, thus increasing the spark gap and frequency of electric discharges, and eventually resulting in an increased MRR. Furthermore, the generation of multiple but finer discharges also leads to fine surface roughness due to the smaller crater size. Other benefits of using CNF in the micro-EDM of ceramics are reduced electrode wear ratio (EWR) and dimensional accuracy, due to the prevention of tool tip concavity, which prevents bi-directional material migration between the electrode and workpiece.

**Figure 14:**
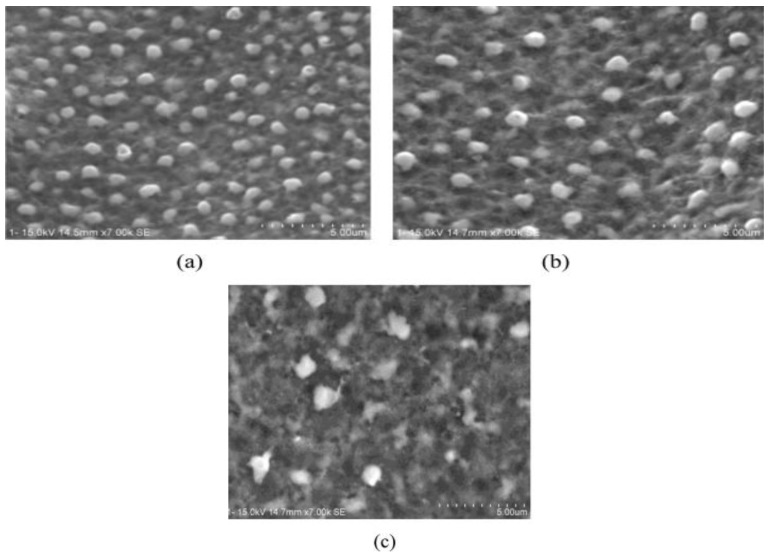
Machined surface at stray capacitance but different levels of voltage with carbon nanofibers addition: (**a**) 60 V (**b**) 80 V (**c**) 100 V. (Reproduced with permission from [110]).

**Figure 15:**
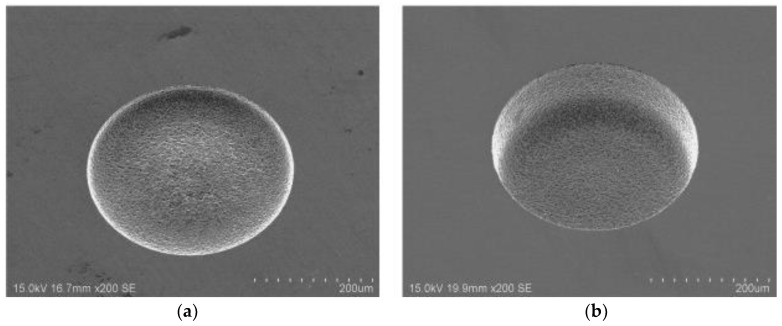
SEM micrographs of micro-holes after machining time of 2 min with ultrasonic cavitation in (**a**) pure EDM oil and (**b**) carbon nanofibers mixed EDM oil. (Reproduced with permission from [111]).

Liew et al. [110] also investigated material migration phenomena by an analysis of tool (tungsten) and workpiece (reaction-bonded silicon carbide) microstructure. Their results suggested the formation of amorphous micro particles due to the deposition of W inside the generated crater. Additionally, tungsten (W) also deposited on other flat-machined parts as a polycrystalline structure containing an inter-diffusion layer. Energy dispersive X-ray (EDX) results of tool surface also indicated possible material migration from the workpiece, dielectric and CNF due to the presence of a carbon element. However, this deposition, which is influenced by machined surface roughness, capacitors, and voltage, can be reduced by addition of suitable amount of CNF. Increase of surface roughness and capacitors enhances this deposition, while increases in voltage reduces the deposition (Figure 14). On another study, Lew et al. [111], incorporated ultrasonic vibration along with CNF assisted micro-EDM and fabricated micro-holes of 10 µm diameter and more than 20 aspect ratios successfully. The addition of ultrasonic vibration on top of CNF aids in debris removal as well as prevention of material deposition by inducing both a stirring and cloud cavitation effect. Figure 15 shows the effect of CNF and ultrasonic vibration on the hole quality. RenJie et al. [112] combined ED-milling and mechanical grinding using subsequent copper electrodes and diamond abrasive tools on SiC ceramics and demonstrated the machining of a large area with better surface roughness. Materials removal is done in two steps: rough machining with the help of ED-milling and fine finishing with the help of a diamond abrasive tool. Their experimental results suggested the use of negative tool polarity and a longer pulse duration for achieving higher MRR and roughness, and lower TWR (tool wear ratio). In addition, increased voltage and discharge current aids in increasing MRR, TWR and roughness. In another similar study, they also demonstrated the effect of machining parameters on surface integrity. It has been found that with the increase of peak current and pulse on time, the crack population and size increase along with the roughness. The tool material diffusion, which can be enhanced by the positive tool polarity and chemical reaction during the EDM process changes the surface composition [113].

Patel et al. [114] conducted an experimental investigation on Al_2_O_3_/SiCw/TiC ceramic composite and developed a regression model for predicting the roughness of the machined surface. According to their investigation, parameters affecting the surface roughness are mainly discharge current, pulse duration and duty cycle, among which pulse on time appears to have a leading affect. The material removal mechanism involved with the ceramics are mainly melting, evaporation as well as oxidation/decomposition at lower energy setting and thermal spalling at higher energy setting, as suggested by the examination of surface/sub-surface topography. Hence, rough machining followed by fine machining offers a better surface quality due to the fine removal of material without causing any surface or sub-surface crack generation [115]. Chang et al. investigated the material removal mechanism associated with ED milling of Al_2_O_3_-based conductive ceramics (Al_2_O_3_ 80%, TiC 11%, WC 9%) and suggested spalling and melting as removal mechanism at low discharge energy whereas evaporation, melting, spalling as removal mechanism at higher discharge energy [116]. Chiang et al. also proposed a mathematical model for predicting MRR, EWR and surface roughness using central composite design and the response surface method. According to the statistical investigation, discharge current and pulse on time significantly affect the MRR of Al_2_O_3_ + TiC [117]. They also proposed a residual modified grey dynamic model RGM(1,3) in order to predict the same performance parameters with a greater accuracy [118]. A similar study was conducted by Lin et al. using a Taguchi based L18 orthogonal array design and optimized input parameters, which was recommended for higher MRR, lower EWR, and surface roughness [119]. Patel et al. also used Grey Relational Analysis (GRA) for optimizing the EDM process of Al_2_O_3_–SiCW–Ti ceramic and emphasized on the leading effect of discharge current and duty cycle on MRR as well as surface roughness [120]. Calignano et al. investigated Al_2_O_3_-TiC ceramic using electro-discharge drilling and successfully fabricated 400 µm hole with 20 aspect ratio. Their investigation suggested that the probable material removal mechanisms during the EDM of ceramics are melting and evaporation. They also proposed a regression model for MRR and EWR as a function of duty ratio and power, which fits quite well with the experimental results [121]. Table 3 provides a summary of conductive ceramics machining using the EDM.

## 5. EDM of Non-Conductive Ceramics

**Figure 16:**
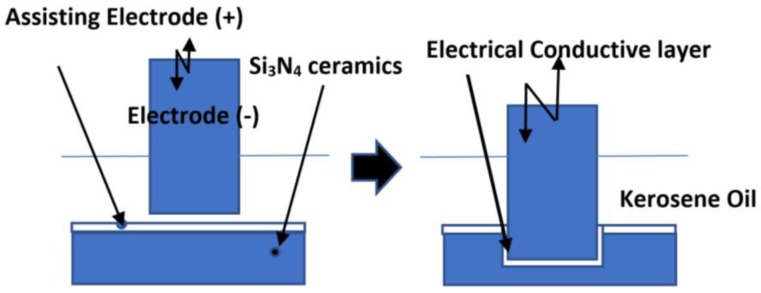
Basic principle of EDM of non-conducting ceramics with an Assisting electrode. Reproduced with permission from [53].

**Figure 17:**
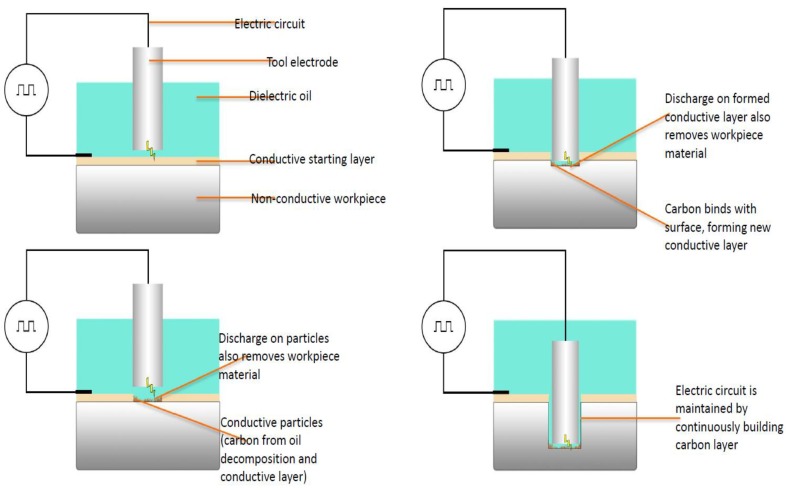
Assisting electrode scheme for machining nonconductive ceramic materials [154].

For the EDM process to be successful, a minimal electrical conductivity of material of 10^−2^/Ωcm is needed [61,142]. Yoo et al. have successfully doped yttrium nitrate (YN) with SiC and showed that the EDM process can be used for machining these ceramics [143]. Nevertheless, it has been reported that doping usually negatively affects the mechanical properties of the ceramics. Apart from this, “Assisting Electrode Method” was suggested in the literature, by Mohri et al. (1996). In the Assisting Electrode method, a conductive layer is applied on the surface of the non-conductive ceramics as shown in Figure 16. Electric sparks cause the generation of high temperature, which forces the molecules of dielectric oil hydrocarbons and workpiece material to crack, which in turn enables the binding of carbon to certain elements of the ceramic. Since the carbon compounds are conductive, new discharges allow the machining of deposited conductive layer together with the workpiece material, which was initially under the conductive layer. The process of particles deposition is shown in Figure 17. By controlling this process, the stable repetition of this sequence of removing the formed layer and deposition of a secondary layer onto the surface is possible. As long as a conductive layer can be formed reliably, the machining is possible [54,62,144]. Mohri et al., also suggested to incorporate a condenser between the tool and the workpiece and strike a balance between long discharge and short discharge in order to increase the MRR and the machining stability [145]. Schubert et al. investigated the machining of zirconia ceramic using this assisting electrode method. It has been reported that for continuation of machining even after the electrically conductive layer is removed, it is essential to use a suitable dielectric and machining parameters [140]. This means that the process is stable when a second conductive layer is rebuilt, which generates a closed electric circuit. The workpiece chosen was ZrO_2_ with Y_2_O_3_ stabilization. The starting conductive layer was silver varnish with 45% silver content, which was applied by a paintbrush. The thickness of this layer was about 20 µm. When using the same parameters for EDM with assisted electrode, the surface roughness obtained in these materials was about two- to three-fold of the roughness of steel. For further studies, it was suggested to conduct investigations aimed to understand the nature of a rebuilt intrinsic conductive layer and the effect of process parameters on the generation of a conductive layer. It was also suggested that the effect of discharge energy and strategy of gap width regulation on the machining time to be further investigated. Schubert et al. [141] conducted another investigation on the micro EDM of two ceramic composites; i.e., Si_3_N_4_-TiN and Alumina Toughened Zirconia (ATZ). The ablation behavior of Si_3_N_4_-TiN doubles the MRR when compared to ATZ. However, this process leads to the decomposition of composite that results in a foamy and porous microstructure. A crucial aspect that must be considered is the formation of the conductive carbon layer. Because of high electrical resistance, especially at a higher machining depth, there are limitations in achievable dimensions. Using a tungsten carbide rod of a diameter of 115 µm, the maximum bore depth achieved was 605 µm and 731 µm for Si_3_N_4_-TiN and ATZ, respectively. The paper concluded that further investigations about the carbon bonding with the ceramics could explain the mechanism and characteristics of the formed conductive layer, such as its conductivity and its crystal structure. Ali et al. (2018) [146] showed that a single spark erosion was not applicable for machining of ceramic in micro-EDM because of random spalling. Especially in the case of the assisting electrode method, it is difficult to create a single spark on a workpiece of nonconductive zirconium oxide (ZrO_2_). For the micro-EDM, the RC generator circuit is suitable because it can be controlled precisely to create lower energy with a shorter discharge [73] (Kunieda et al. 2005). In this work, a nonconductive ceramic (ZrO_2_, Al_2_O_3_, Si_3_N_4_ and SiC) was successfully structured by using the assisted electrode method and by manipulating the micro-EDM process parameters such as voltage, capacitance, and polarity. It is also shown that capacitance and resistance have a significant effect on the charging and discharging duration. However, at higher capacitances the formation of a carbonic conductive layer on the machined surfaces was not stable. It was observed experimentally that the MRR was lower than the theoretical values because of the longer pulse-off time and the effect of different ineffective pulses such as short circuit, delayed discharge, immature discharges, and arching. From this investigation, it was found that capacitance significantly affected the formation of a pyrolytic carbon layer. However, the voltage mainly controlled overall MRR. Zeller et al. (2014) [147] investigated the micro-structuring of a non-conductive silicon carbide using the EDM. In this experimental study, the workpiece was non-conductive sintered silicon carbide (SSiC) with an assisting electrode applied by a screen printing process. The machining process was unstable at the beginning and it required the adjustment of process parameters and the tool geometry. The authors found that the cause of unstable machining might be due to the excessive generation of carbonized products. The modification of two parameters; i.e., current and frequency was done to reduce the amount of carbonized products. The average thickness of 24 µm with a standard deviation of 1.6 µm was measured for carbonized products/coatings. The modification of tool geometry was done to improve the flushing conditions. The process was successful up to the maximum machining depth of 500 µm. Srivastava (2016) [148] studied the EDM of ZrO_2_ with copper, brass and graphite tool electrodes. A new lacquer-based Assisting Electrode (AE), which enabled the stable erosion process was applied by screen print or Doctor Knife techniques. For doctor knife coated AE, the thickness of the layer was 356 μm with a standard deviation of 71 μm while for the screen printing the layer was 30 μm with a standard deviation of 3 μm. It was concluded that the copper electrode was suitable for erosion depths smaller than 500 μm while the tungsten copper was more suitable for depths larger than 1 mm. Sabur et al. (2013) [8] studied the machinability of the ceramic ZrO_2_ using the assisting electrode method of die-sinking EDM. Bores with depths of 1 mm, 2 mm, 4 mm were machined on the workpiece. On the machined surface, the pyrolytic carbon layer was created continuously by using copper tool electrode with a negative polarity. The kerosene was used as a dielectric and an adhesive copper foil was used as assisting electrode. The mechanism of material removal in EDM of insulating ceramics is mainly spalling, which was confirmed by the scanning electron microscope (SEM) images showing that there are several cracks due to spalling. This paper shows that at lower input powers, EDM of ZrO_2_ cannot be processed. The minimum power needed for the stable formation of pyrolytic carbon layer to achieve a low MRR was 1.2 KVA. It was also observed that at the initial stage, an increase of input power resulted in an increase of MRR. This trend is true for the input power range of 1.1 KVA–1.3 KVA. In the range from 1.3 to 1.4 KVA, an increase in MRR was lower. In another study, the assisting electrode method was used in machining of Si_3_N_4_, SiC, AlN, and ZrO_2_ [149]. The thickness of intrinsic electrically conductive adhered layer varied from 30 to 50 µm, which consisted of three regions. The lower layer consisted of zirconium and copper, the middle layer consisted of zirconium and carbon and the upper layer consisted of zinc and carbon. Carbon came from the decomposition of oil dielectric. Zinc and copper came from the melting of the tool. The results suggested that the thickness of an intrinsic electrically conductive layer increased with the increase of open circuit voltage while bending strength decreased for a high value of open circuit voltage. By using this method, it was possible to achieve slit machining and hole machining of micrometers size on the non-conductive ceramics.

In the investigation of Banu et al. (2014) [150] the MRR and the recast layer hardness after micro-EDM of non-conductive zirconia ceramic were analyzed. As an assisting electrode, the gold coating and copper adhesive foil were chosen. The results show that the MRR is better when the gap voltage is low, and the tool electrode is coated with carbon elements, which are generated from the decomposition of polymer chains during the spark. The recast layer increases when the rotational speed increases. This occurs because the debris and carbon elements cannot be removed from the machining area and they are quenched rapidly, so the recast layer is formed. Yeakub et al. (2016) [151] investigated the micro-EDM of non-conductive zirconia with titanium carbide powder. In this study, two parameters were varied: capacitance and gap voltage. Using two controlled factors for MRR, a Taguchi L9 orthogonal array was used. The adhesive copper foil was used as an assisting electrode. A copper lid was clamped to the workpiece on top of the copper foil. This lid was used to trap powder additive and carbon, which helped to obtain the stable machining process. In this study, it was shown that a customized setup could achieve higher MRR than a standard setup. Based on the ANOVA analysis, it was found that the factor that affected the MRR mostly was the capacitance. Ferraris et al. [152] have conducted an experimental investigation on the micro-EDM behavior of ZrO_2_ and Al_3_O_2_ non-conductive ceramics, with a secondary conductive phase addition within the insulating ceramic matrix. The micro sized TiCN particles were uniformly distributed within the ceramic matrix by using a pulsed electric current sintering. A two-level Taguchi L8 orthogonal array was used as an investigating methodology to examine the effect of the input parameters on surface roughness, material removal rate, and relative tool wear of the two materials. The machining process was controlled by the following non-standard parameters: current (A), open voltage (V), frequency (Hz), energy (code), and width (µs). The results suggested that in comparison to ZrO_2_-TiN, Al_3_O_2_-TiCN shows lower surface roughness, which is due to the higher amount of secondary conductive phase. But the durability of the machining may be affected since the generation of subsurface cracks was also observed. The process behavior for both materials was similar to steel. However, the tool wear was different and up to four times lower than steel. Singh and Sharma (2016) [153] carried out an experimental investigation on the powder mixed EDM of cobalt-bonded tungsten carbide (WC-Co). The tool diameter was 17 mm and made of electrolytic copper. For this experiment, graphite powder mixed dielectric was used. The average grain size of the graphite powders was 55 µm, and the powders were mixed in the dielectric at a concentration of 15 g/L. Three dielectric fluids were investigated, namely, kerosene oil, distilled water and EDM oil. It was found that MRR and TWR are opposite in nature and both require different requirements to be improved. It was concluded that a multi-objective optimization should be done, however, there was also a need to substitute the dielectric oil with the one with less consumption and minimal production of harmful effects.

**Figure 18:**
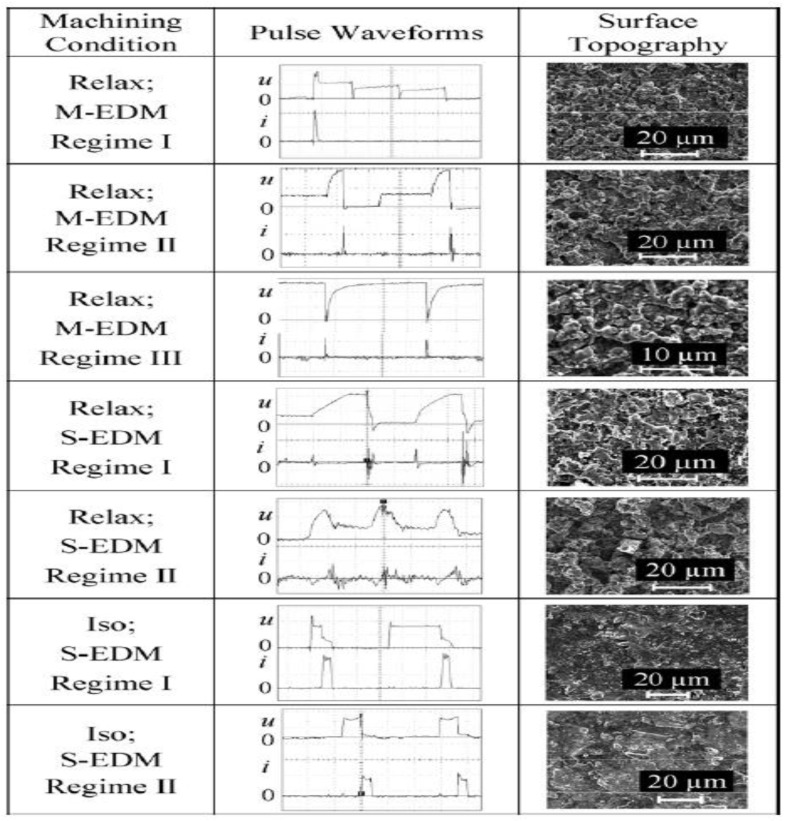
Pulse shapes and corresponding surface topographies. (Reproduced with permission from [140]).

**Figure 19:**
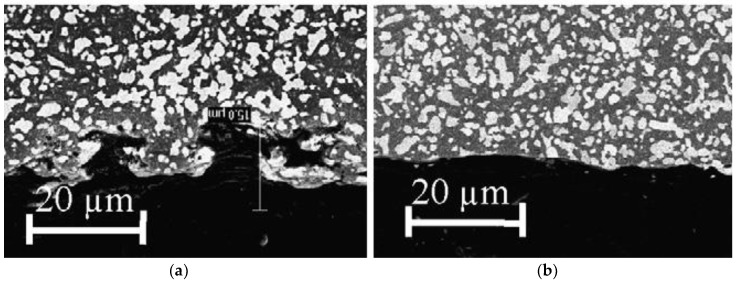
Cross-section views of samples machined by S-EDM: (**a**) relaxation pulse and (**b**) iso-energetic pulse. (Reproduced with permission from [140]).

Sabur et al. (2013) [8] investigated the micro-EDM of ZrO_2_. Since ZrO_2_ is nonconductive, a conductive adhesive layer of copper was applied on the surface of the workpiece. Experiments were performed by varying the voltage, capacitance, and electrode rotational speed. The Taguchi method of experiment was used to correlate these variables and create a mathematical model of surface roughness. The paper concludes that the most significant parameters for surface roughness are voltage and capacitance and that increasing these parameters results in an increase of surface roughness. It is also suggested that further experiments at higher capacitance values are needed to determine the effect of parameters on surface roughness. In the study of Gadow et al. (2016) [155,156], the ZTA-TiC (zirconia toughened alumina-titanium carbide) ceramic, which has high strength and hardness with moderate fracture resistance and good ED-machinability, was investigated. The machinability was assessed by performing die sinking EDM with fixed parameters: starting voltage of 150 V, discharge duration of 5 µs and a discharge current of 10 A in ionoplus medium. Wire-EDM with Zn-coated brass (diameter = 0.1 mm) was performed using CUT1000 Oiltech wire EDM machine. From the mechanical and electrical properties of hot pressed ZTA-TiC, it was observed that the hardness and the young’s modulus were comparable to ultrafine alumina. Also, the value of high strength and moderate resistance to fractures were obtained after EDM. It was shown that a surface machined by die sinking EDM did not have a foamy or glassy layer and that there was no damage to the bulk material. Liu et al. [140] investigated the influence of type of pulses on the machinability of Si_3_N_4_ ceramic in EDM. Relaxation pulses during the micro-EDM resulted in a better roughness profile with higher machining speed, whereas during sinking EDM, rougher surface with higher machining speed was observed as shown in Figure 18. The iso-energetic pulse during S-EDM gave a finer surface with less machining time compared to the relaxation pulse, however higher tool wear was reported in this case. The material removal mechanism was influenced by the pulse type and pulse duration. For shorter pulse duration (relaxation pulse), the leading material removal mechanism was found to be decomposition as well as oxidation. Foamy as well as porous structure was a consequence of this chemical reaction, which releases nitrogen gas and reduces tool wear while increasing machining speed. For longer pulse duration (iso-energetic pulse) during S-EDM, melting became the dominant material removal mechanism due to the presence of higher energy. Therefore, the relaxation pulse generator resulted in a higher MRR while iso-energetic resulted in a smoother surface (Figure 19).

**Figure 20:**
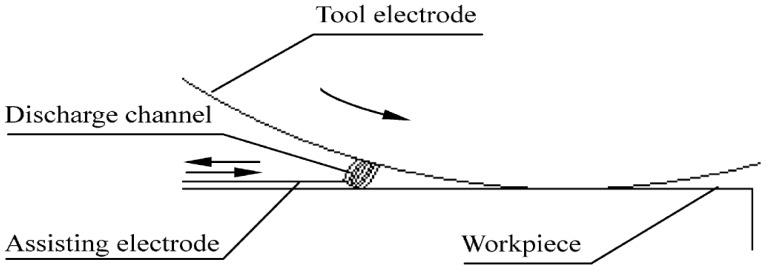
Principle of ED milling. Reproduced with permission from [57].

**Figure 21:**
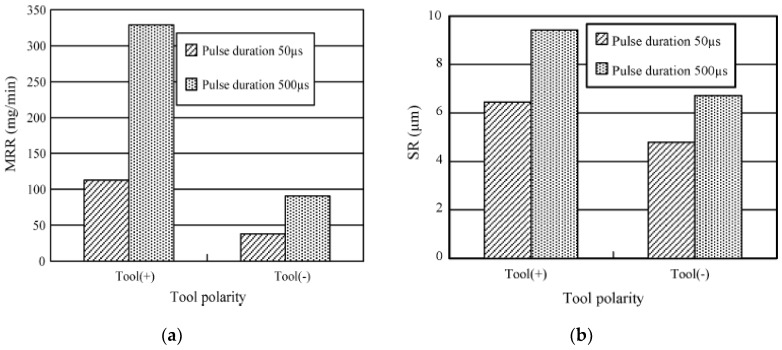
Effect of tool polarity on (**a**) MRR (**b**) surface roughness. (Reproduced with permission from [57]).

Liew, Yan and Kuriyagawa (2013) [157] investigated the micro electric discharge machining of reaction-bonded silicon carbide with the assistance of a carbon nanofiber to examine the material removal rate, surface finish, electrode wear ratio, surface damage, spark gap, and surface topology. It was observed that higher carbon nanofiber concentration improved the material removal rate, electro discharge frequency, discharging gap and reduced electrode tip concavity, and electrode wear. Results showed that the addition of the carbon nanofibers leads to the generation of several discharging paths from a single input pulse indicating that electro discharge characteristics were improved multiple times. This occurred because the discharging energy was dispersed by the nanofibers. Experiment data showed that in case of EDM with added carbon nanofiber, the MRR rapidly increased. The maximum MRR was at a concentration of 0.17 g/L carbon nanofibers. However, there is a trend of the MRR reduction when concentration of nanofibers is higher than 0.17 g/L. Such a trend is consistent with another investigation conducted by Jahan et al. [158]. The electrode wear ratio was found to be higher with pure dielectric fluid because ions produced from dielectric fluid ionization hit the tool electrode with high energy and high momentum. Therefore, the higher the concentration of carbon nanofibers, the lesser the electrode wear ratio. Also, the carbon nanofibers addition causes a larger spark gap and the flushing of debris become easier. If the debris cannot be removed effectively, the probability of short circuit occurrence between electrode and workpiece increases. Such a situation is not favorable because it leads to a low MRR. Regarding the surface roughness, it tends to increase as the concentration of the carbon nanofibers increases and therefore there is an optimum concentration, which is 0.02 g/L for time controlling and 0.06–0.1 g/L for depth-controlling conditions. Due to the limitation associated with EDG and EDM; ED milling (Figure 20) was developed by Liu et al. [57] to machine a larger surface using a thin copper sheet as an assistive layer on a nonconductive Al_2_O_3_ ceramic. For this process, a copper sheet and steel electrode are connected with the negative and positive terminal, respectively, and a water-based fluid is flushed between the two electrodes. As the tool electrode gets closer to the assistive electrode, sparks initiate when the distance between two electrodes reaches the gap discharge. During this experiment, it was observed that positive tool polarity contributed to a higher MRR and higher roughness compared to the negative tool polarity (Figure 21). This can be due to the higher pulse on time of ED milling process that gives enough time to the heavy mass positive ion to accelerate and collide with the workpiece surface with a high impact energy. It was also reported that the tool wear ratio was higher for the negative tool polarity. In addition, the increase of tool rotation, feed rate and peak voltage; both the MRR and surface roughness increased.

**Figure 22:**
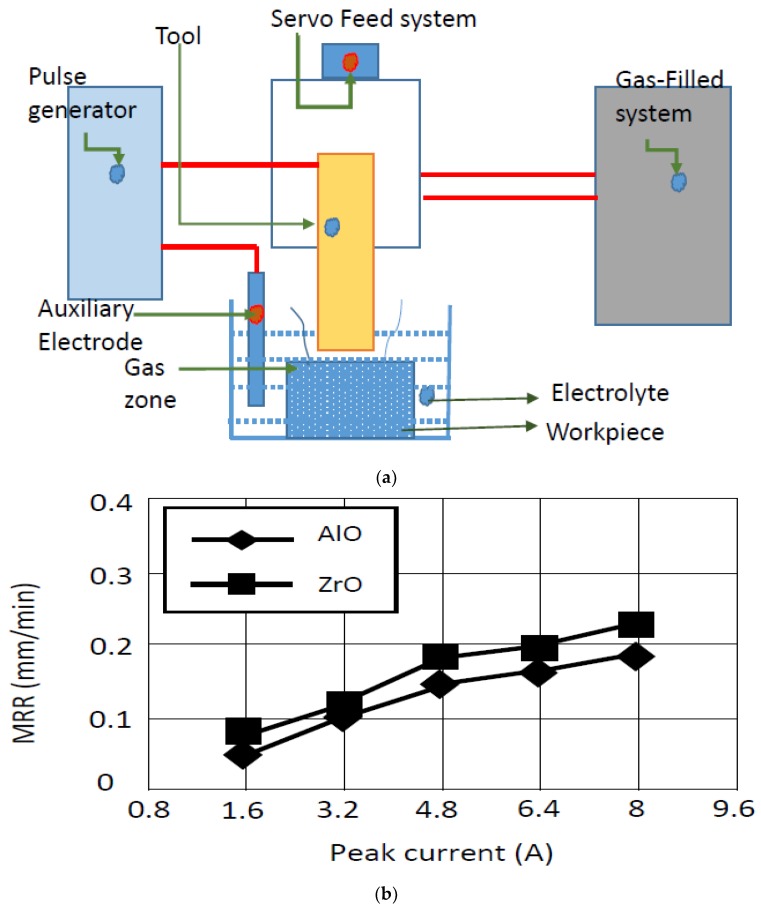

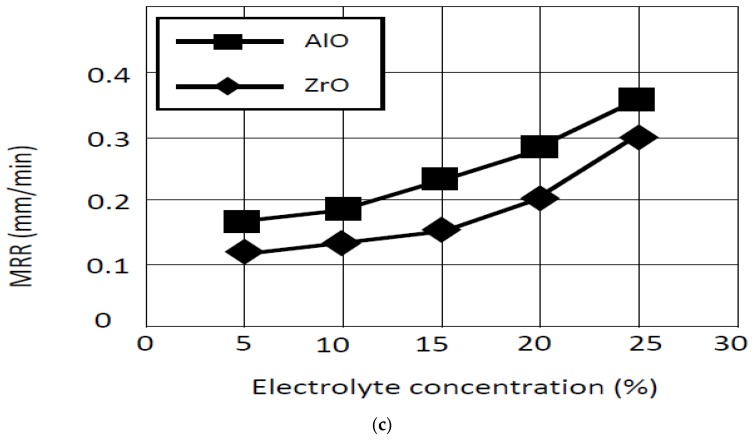
(**a**) Principle of gas-filled electro discharge and electrochemical compound machining (GFEECM), (**b**) Effect of peak current, (**c**) Effect of pulse duration on the MRR. (Reproduced with permission from [159]).

**Figure 23:**
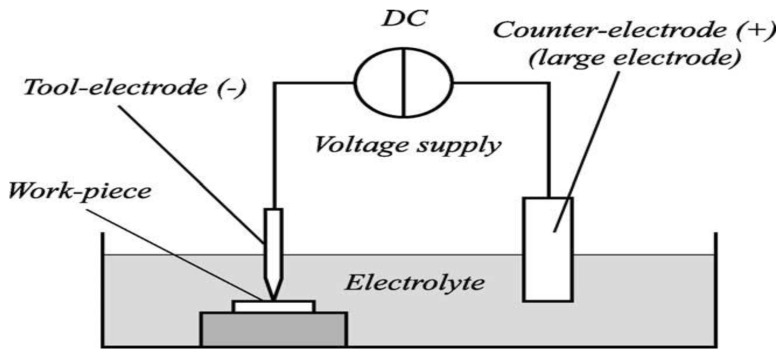
Basic cell configuration in electro-chemical discharge machining. Reproduced with permission from [162].

According to the study of Liu et al. [159], gas-filled electro discharge and electrochemical compound machining (GFEECM) for Al_2_O_3_ ceramics exhibited relatively less electrode wear, better shape accuracy and roughness. The principle of GFEECM is shown in Figure 22, where the side and bottom face of the tool are covered with the gas coming from the gas-filled system. A voltage is applied across the auxiliary electrode and the tool electrode helps in developing an electric field, which eventually reaches the breakdown strength of the ceramic and the gas was able to exist into the gap. This initiates a plasma channel of high temperature and pressure. Once the material is removed, the distance between tool and workpiece is increased causing electrolytic fluid to enter into the gap [159]. These results suggest that NaOH electrolytic can be used for Al_2_O_3_ and ZrO_2_, but NaNO_3_ is not suitable for the EDM machining of Si_3_N_4_. An increase of electrolyte concentration as well as the peak current can improve the MRR (Figure 22b,c). Zaripov and Ashurov (2011) [160] also reported about a process that combined EDM and electrochemical processing (Figure 23) for a composite non-conducting material. In this paper, the flow of current in an electrolytic media and the processing of a composite material was analyzed. It was presented that the machining of nonconductive materials can be done in an electrolytic medium in the mode of formation of a plasma sheath near the cathode. It was suggested that the combination of EDM erosion and electrochemical machining includes ultrasonic components.

In the investigation made by Li et al. (2012) [161], a new method for EDM of non-conductive ceramics was developed. The new method, named double electrodes synchronous servo electrical discharge grinding (DESSEDG), integrated the advantages of EDM and grinding, which achieves high efficiency precision machining. In this technique, a conductive grinding wheel rotates on the surface of a workpiece while a sheet electrode is fed to the contact point of the workpiece and wheel. The discharges generated a high temperature and pressure plasma channel which were exerted to the workpiece surface for the material removal. Then the eroded layer was machined by grinding of the conductive wheel. The advantages of DESSEDG include high efficiency, low machining cost and environmental pollution-free. Table 4 provides a summary of the EDM of non-conductive ceramic. Table 5 provides a summary of machining performance parameters during ceramic machining

## 6. Current Challenges and Future Research Direction

Despite their high electrical resistivity, ceramic materials can be machined using the electro-discharge machining process (EDM) either with the use of an assistive electrode or by doping them with the secondary conductive phase. Since the secondary conductive phase creates some issues related to the degradation of ceramics properties, the amount of the conductive phase must be controlled in order to make sure the non-alteration of the ceramic properties occurs. The machining accuracy as well as the stability and electrode wear ratio depend both on the type of assistive electrode usage and the machining parameters. As can be seen in the literature, the long discharge duration during EDM of an insulting ceramic results in a carbonized layer generation on the surface, thus assisting the material removal with normal discharges. The material removal mechanisms related to the insulating ceramics are found to be mainly thermal spalling, melting/evaporation, whereas oxidation or decomposition, as well as melting are dominant for the conductive ceramics, which also can be affected by the dielectric usage and discharge energy. Moreover, carbon nanotubes, when added to dielectric, enhance the machinability of the non-conductive ceramics. For the optimum MRR and reduced sub-surface damage; higher current, long pulse on time along with short pulse off time need to be avoided. In addition, both the MRR and surface roughness are influenced by the pulse energy and pulse on time. Moreover, the shape, polarity and abrasives associated with the tool also influence the process capability significantly. For the higher MRR and lower TWR, longer pulse duration and negative polarity are required, whereas smaller pulse duration aids in a better surface roughness.

Although the research studies on the machining of ceramics using EDM based techniques are rapidly increasing, an improvement of the overall machining process is still essential. A significant amount of research questions need to be answered before the EDM process becomes an effective manufacturing process for machining both the conductive and non-conductive ceramics. This section summarizes some of the major challenges reported in the literature and the future research scopes in the areas of the EDM and micro-EDM ceramics.

- One of the important criteria for successful machining of ceramics is the selection of an assisting electrode. The challenges faced by the current researchers include the selection of coating material, appropriate coating thickness, and ways of creating these coatings. Since there is a wide variety of assisting electrodes, it is difficult to effectively choose the kind of assisting electrode that would fit a particular area of application. No specific criteria based on the electrical conductivity or other electro-thermal properties are presented in the literature. Moreover, studies on investigating the effectiveness of various coatings for a single material and the selection of an optimum coating originated on physics based reasoning are missing. Hence, future studies should focus on establishing the guidelines for the selection of an assisting electrode based on material properties, and the structure of the ceramics and the underlying physics.
- Another important challenge of EDM of ceramics is the need for a modified pulse generator specifically designed for conducting EDM on ceramics. Several studies have reported pulse duration as one of the most influential parameters for successful machining. Hence, there is also a need to develop a control algorithm for the pulse generator that would precisely control pulse duration. This parameter significantly affects the intrinsic conductive layer, which is important in machining non-conductive ceramics [53]. The growth of the intrinsic conductive layer with the increase of pulse duration can be modeled, which would provide proper explanations behind the mechanism of growth of the conductive layer, and if any other external factors/parameters play a role in the process.
- So far, the majority of the research studies on EDM of ceramics focused on feasibility and performance studies by carrying out experimental investigation. The major challenge is to understand the physics of the process and to develop physics based modeling for the EDM of ceramics. Considering the physics of processing for non-conductive ceramics, numerical models should be developed, as well as existing models should be improved. The physics based modeling should address the mechanism of formation of intrinsic conductive layer, and how that influences the material removal mechanism and further advances the machining process. The analytical (physics based) and numerical (Finite Element Method based) modeling should focus on the EDM induced surface modification, crack formation, and mechanical property changes of the ceramics. Thermal fractures should be considered during the modeling to understand the crack formation in the surface and sub-surfaces.
- Analytical and numerical modeling of the material removal mechanisms during EDM and micro-EDM of ceramics is of prime importance to broaden the application of EDM usage in ceramic machining. This is one of the major challenges faced by the current researchers due to the complex nature of the material removal involved in EDM. Several material removal mechanisms have been discussed in the literature, such as, melting and evaporation, thermal spalling, fusion and vaporization, oxidation and decomposition. Hence, it is important to investigate whether the material removal mechanism in EDM/micro-EDM of ceramics is significantly different from the EDM of conductive metals and, if different, then establishing the analytical and/or numerical model of the material removal mechanism during EDM/micro-EDM of non-conductive ceramics. It is also important to investigate whether the material removal mechanism has any correlation with thermos-electrical or thermos-physical properties of ceramics.
- Few studies focused on predictive modelling to understand the effect of EDM on the machined product [189]. It is extremely imperative to evaluate the post-machined characteristics of components to analyze the effectiveness of the process on machined ceramics. Future studies may include the measurement of residual stresses, hardness and the methodology can be similar to the studies that were done for metals such as in [190] or [191]. In addition, the phase transformation and the changes in the crystal structures of the ceramics due to the EDM process need to be investigated in future research.
- One of the major challenges of EDM and micro-EDM of ceramics are slow machining rate, post-processing requirement to improve the surface finish, and low throughput. Therefore, the future research trend should focus on solving the associated problems either by developing newer hybrid machining processes or by incorporating novel ideas to improve the existing process and creating new processes for machining ceramics. There have been few research studies on nanopowder mixed EDM, ultrasonic vibration assisted EDM, CNT or graphene mixed EDM of ceramics. However, a fundamental understanding and modeling of those hybrid processes are still missing.
- Finally, most of the studies have focused on the feasibility testing and machinability studies of various ceramics. Very few studies have focused on machining parts, components, or features of ceramics using EDM/micro-EDM for real life applications in the industries. The challenges that the current researchers will face is the broadening of applications by machining industrial grade parts and components. In order to establish the EDM/micro-EDM as a process of choice to the industries, extensive research on the industrial applications of the process is needed. Therefore, future studies should focus on machining of high aspect ratio micro-holes, complex 3D micro-features, and 3D functional parts on ceramics.

## Figures and Tables

**Table 1:** Application & properties of ceramics materials [2].

Applications	Performance Properties	Ceramics
Wear parts: seals, bearings, valves, fuel nozzles, aerospace industry, cutting tool inserts, automotive brakes, prosthetic products, piezoceramic sensors, biomedical implants, mold-dies [44], heat engines [45], next generation computer memories [46].	High hardness, lower friction, high thermal conductivity, high stiffness, and low density [47].	SiC, Al_2_O_3_
Cutting tools, gas turbine impeller manufacturing [48].	High strength, high hardness, thermal shock, and oxidation resistance	Si_3_N_4_
Heat engines: Diesel engines components, gas turbines.	Thermal insulation, high temperature strength, Fuel economy, exceptional high fracture resistance [49], good corrosion resistance [8].	ZrO_2_, SiC, Si_3_N_4_
Medical implants: Hip joint, teeth, other joints.	Biocompatibility, machined surfaces’ bond to tissue, corrosion resistance.	Hydroxyapatite, Bioglass, Al_2_O_3,_ ZrO_2_
Ballistic applications, shielding in nuclear fission reactors, bearings, dies, cutting tools, extrusion nozzles, seals and rings [50].	Excellent hardness, wear resistance, fracture toughness properties Low density, high compressive strength, high elastic modulus.	B_4_C
Construction: highways, bridges, buildings.	Improved durability, low overall cost.	Advanced cements & Concrete

**Table 2:** Properties of different ceramics [63,65].

Properties		Aluminum Oxide (Al_2_O_3_)	Silicon Carbide (SiC)	Silicon Nitride (Si_3_N_4_)	Partially Stabilized Zirconia (PSZ)	Silica, Fused (SiO_2_)
Transverse rupture strength (MPa)		140–240	100–750	480–600	620	-
Compressive strength (MPa)		1000–2900	700–3500	-	-	1300
Elastic modulus (GPa)		310–410	240–480	300–310	200	70
Hardness (HK)		2000–3000	2100–3000	2000–2500	1100	550
Poisson’s ratio, υ		0.26	0.14	0.24	0.30	0.25
Physical properties	Density (kg/m^3^)	4000–4500	3100	3300	5800	-
	Thermal conductivity (W/m/K)	8.4	83.6	25	2	-
	Thermal expansion coefficient (m/m/K)	9.0 × 10^−6^	4.3 × 10^−6^	3.2 × 10^−6^	10.6 × 10^−6^	-
	Specific heat (J/kg/K)	1040	1040	710	543	-

**Table 3:** Summary of EDM of conductive ceramics.

Ceramics Type	Composites	Type of Operation	Material Removal Mechanism	Remarks
Aluminum based	Al_2_O_3_-TiC composite	ED-Drilling	Combined melting and evaporation	Crater diameter is not affected by pulse duration but increases with pulse power [121].
	Al_2_O_3_–SiCW–TiC	Diamond-G, EDM, EDG		EDG offers 50 times higher MRR and 4 times less roughness than EDM. EDG also produces 4.5 times better surface than diamond grinding [122].
Zirconia based	ZrO_2_-WC (40%)	W-EDM	Full melting and evaporation	MRR increases with pulse duration and decreases with pulse interval for both coarse and fine ZrO_2_-WC [123].
	ZrO_2_ composite	W-EDM	Full melting and evaporation.	With pulse duration MRR increases and WC based ceramics exhibits better roughness [124].
	ZrO_2_-TiN	W-EDM	Melting, evaporation & Chemical decomposition	With the increased number of finishing cut, roughness reduces for every cutting dimension, however bending strength does not vary much [125].
	ZrO_2_-TiN	W-EDM	Melting, evaporation & Chemical decomposition	Finish cutting seems not be related with flexural strength [126].
	SiC, B_4_C, Si_3_N_4_-TiN	M-EDM, S-EDM		Micro-EDM provides better performance for MRR and surface quality compared to S-EDM [127].
Silica based	SiSiC	S-EDM		First order model proposed was satisfactory for Sm (mean spacing of profile irregularities); pulse time and duty cycle increase the mean spacing distance while voltage does opposite [128].
		S-EDM	Melting and evaporation	For optimum MRR and to avoid subsurface damage, high peak current, long pulse on with short pulse off combination needs to be avoided [129].
	Si, SiC	Multi W-EDM		Straight and uniform kerf can be achieved by using brass coated steel wire having track shaped section and increased wire tension, this process also suffers less vibration [130].
	SiC Single ingot	EDM		EDM causes low surface damage compared to diamond saw cutting [131,132]. Kerf loss and roughness achieved are much less for EDM. Using maximum cutting speed of 0.8 mm/min and 50 μm wire, 2 inches ingot can cut within 7 h [133].
	SiC Single ingot	Multi-discharge EDM coring method;		Multi-discharge EDM with 6 electrodes offers either simultaneous or sequential discharge during single pulse duration. With improved discharged frequency, surface integrity and machining efficiency gets better [134].
	TiN/Si_3_N_4_	Sinker-EDM		With current, electrode wear ratios increases and brass experiences higher wear compared to copper [135].
	TiN/Si_3_N_4_ (37.5 & 40% TiN)	WEDM		MRR increases with the aid of silver layer and depends on the relative position of wire and clamping [136].
	Si_3_N_4_ composite	EDM		With the addition of a proper secondary conductive phase, EDM of complex shape can be generated [137].
	Si_3_N_4_/CNTs	EDM		EDM process offers high MRR, low roughness as well as tool wear compared to Si_3_N_4_/TiN. With voltage, MRR, TWR and roughness show increasing trends [138].
	Si_3_N_4_–TiB_2_	S-EDM W-EDM		40% addition of TiB_2_ makes the ceramics conductive enough to be machined by EDM [139].
	Si_3_N_4_–TiN	M-EDM, S-EDM	melting, decomposition and oxidation	Iso-energatic pulse with S-EDM offers better surface and high tool wear whereas relaxation pulses offers contrary results [140].
	Si_3_N_4_–TiN	WEDM	Melting, evaporation, thermal spalling	MRR increases with increases of power and decreases with increases of pulse off time [141].

**Table 4:** Summary of EDM of Non-conductive ceramics.

Ceramics Type	Composites	Type of Operation	Material Removal Mechanism	Remarks
Al based	aluminum oxide (Al_2_O_3_) ceramic	ECDM	Electrochemical (EC) reaction and electrical spark combined	Higher MRR and dimensional accuracy can be attained by using 80 V and 25% NaOH electrolyte [163].
		AE-EDM	Melting, dissociation, Evaporation	Copper-infiltrated-graphite (Poco-EDM-C3) outperforms copper; graphite (Poco EDM-3) in term of MRR and EWR, better surface roughness [164].
		AE-EDM	Melting, Evaporation, Spalling [165]	Single discharge crater volume increases with voltage and capacitance increment, while it decreases with increasing resistant as well assistive electrode thickness [166].
		Electrical discharge (ED) milling		Higher flow velocity of dielectric increases MRR and offers improved surface roughness [167].
		ED-milling		Simulation results of thermal eroding shows agreement with experimental results [168].
		ECDM		Pulsed DC reduces the chance of crack formation compared to smooth DC and abrasive electrode increases MRR [169,170].
		Electrochemical spark abrasive drilling		Increase in voltage and temperature of electrolyte can enhance machining performance [171].
Zr based	Zr_2_O_3_, SiC, Si_3_N_4_	AE Wire EDM	flake by flake	ZrO_2_ performs well in terms of material removal [172].
	Zr_2_O_3_	AE WEDM	Chemical decomposition	For pulse on time of 20 μsec and high machining speed, low roughness can be reached [173,174].
			Melting, Spalling, crack formation	Presence of monoclinic zirconia, suggesting the conversion of ZrC to Zr_2_O which can be prevented by higher temperature oven process with 10 K/s active cooling [175].
	ZrO_2_-Y_2_O_3_	AE Sinking EDM		Copper porous electrode with 85% density provides maximum MRR. Volumetric wear ratio reduces with the increase of tool density [176].
	Zr_2_O_3_	AE ED-milling		Discharge pulses have shorter peak but longer duration for ceramics [177].
	Zr_2_O_3_	AE Wire EDM;		MRR increases with peak current and pulse on time [178]. Model MRR and surface roughness was proposed [179].
Si based	Si_3_N_4_	AE WED-milling		Discharge duration & duty factor both increase MRR & surface roughness [53].
		AE Die sinking EDM		Voltage enhances electrode wear ratio but, increased diameter reduces this ratio [180].
		Ultrasonic assisted AE-EDM		MRR increases twice compared with Assistive EDM, however roughness increases due to vibration [181].
	Si_3_N_4_	AE-WEDM		Using higher current, 100 mm thick plate was successfully cut where straightness as well as roundness value reached to 12 and 17 μm [182].
	SiC	ED-Milling		Positive tool polarity results in better MRR and low EWR [183].
	Si_3_N_4_	WEDM		Conductive layer has much effect on thermal transmission in radially than in crater depth direction [184].
	Si_3_N_4_	EDM		Predictive accuracy seem high and convergent is present [185].
	Si_3_N_4_	ECDM		Mathematical model for MRR, Radial overcut, heat affected zone suggested leading effect of voltage [186].

**Table 5:** Summary of EDM performance characteristics for Conductive/non-conductive ceramics.

Performance Parameters	Ceramics Type	Type of Operation & Parameters	Remarks
Material removal rate (MRR)	Si_3_N_4_-TiN and Alumina Toughened Zirconia (ATZ)	Micro EDM, Open circuit voltage, discharge type	The ablation behavior of Si_3_N_4_-TiN enables 200% of MRR compared to ATZ [187].
	Zirconium oxide (ZrO_2_)	AE micro-EDM Polarity, flushing, feed rate, gap voltage, and tool electrode, rotational speed	Capacitance significantly affects the formation of pyrolytic carbon layer, however, MRR is mainly controlled by voltage. Experimental MRR was lower than theoretical values [150].
	zirconia (titanium carbide powder mixed with the kerosene)	AE M-EDM, Gap voltage, capacitance	The factor, which affected the most to MRR, was capacitance. 86 V and 1.0 nF are optimum for reaching maximum MRR [188].
	ZrO_2_	AE die-sinking EDM Pulse on time, pulse off time, input power, negative polarity	Mechanism of material removal is mainly spalling. The minimum power needed for the stable formation of pyrolytic carbon layer with low MRR is 1.2 KVA [8].
Surface Roughness	ZrO_2_ and Al_3_O_2_ with secondary conductive phase TiCN	Micro-EDM Current, open circuit voltage, Energy, frequency, pulse width	In comparison with ZrO_2_-TiN, Al_3_O_2_-TiCN show lower surface roughness due to higher amount of secondary conductive phase [152].
	ZrO_2_	AE die-sinking EDM Voltage, capacitance, RPM	Significant parameters for surface roughness are voltage and capacitance and that increasing these parameters results in increase of surface roughness [8].
Dimensional accuracy	Sintered silicon carbide	AE method of micro- EDM, Current, frequency, tool geometry	The adaptation of current and frequency was performed to reduce the carbonized products and adaptation of tool geometry to improve flushing conditions [147].
	Si_3_N_4_, SiC, AlN, and ZrO_2_	AE-WEDM Open circuit voltage	Thickness of intrinsic electrically conductive layer increased with the increase of open circuit voltage [149].
	Al_2_O_3_	Double electrodes synchronous servo electrical discharge grinding (DESSEDG)	The advantages of DESSEDG include high efficiency precision machining, low machining cost and environmental pollution-free [161].
Tool wear	SiSiC	Die-sinking EDM, Discharge current, open gap voltage, discharge duration	Overall EWR is about 30% for both rough and semi-rough condition [129].
	Al_2_O_3_	AE-EDM	Copper electrodes experiences higher EWR compared to EDM-C3 and EDM-3. EWR [164].
			Lower RWR can be achieved with negative tool electrode. Tool wear decreases with decreasing capacitance and increasing current limiting resistance [166].
